# An Explanation

**Published:** 1876-06

**Authors:** 


### *AN EXPLANATION.*

We are almost daily in receipt of letters asking us to procure for the writers the formula for "Van Deren's" Cholera Infantum Remedy, and "Nelaton's Suppository" for Constipation and Piles. We have sent some of these letters to Hall & Ruckel, No. 218 Greenwich Street, New York, and asked them to reply. They have done so, and this is in substance what they say. "First, that they are too well and too favorably known to render themselves liable to the suspicion that they would manufacture and vend any medicine not entirely pure, or that would be liable to do harm. Second, that these medicines are manufactured under the careful supervision of an experienced physician and chemist, from the best materials to be obtained, and that the quantities are proportioned with the greatest accuracy; that it would be impossible for a retail druggist to compound from either formula an article that would compare with those they manufacture, and hence the reputation of their remdies would suffer. Third, that the retail price (50 cents) is less than they could be furnished for by the retail druggist, had he the facilities for properly making them. Lastly, that they withhold the information asked for, from motives of humanity toward the thousands of sufferers who have been, and are to be, relieved by their use, and not by fear of competition in their manufacture."

We believe that every fair minded person will admit the force of these reasons, and would gladly join in a request that the formula should not be published. Every physician remembers the many disappointments he has experienced on account of the bad quality of drugs frequently furnished by retail stores; when, for example, quinine is prescribed for intermittent, with a confident promise of improvement in the symptoms, to find them worse at the next visit. We know then that the quinine must have been worthless. But, usually, detection of adulterated drugs is not so easy, and what should have done good may do positive injury. Some compound medicines, entirely harmless, may be changed into rank poisons by a simple change of their proportions.

In view of these facts we indorse the decision of Messrs. Hall & Ruckel, and trust they will adhere to it.

